# Leptin receptor rs1137101 polymorphism and altered leptin-sOb-R axis contribute to type 2 diabetes risk in Gujarat population

**DOI:** 10.3389/fendo.2026.1693265

**Published:** 2026-01-28

**Authors:** Nishant Parmar, Nirali Rathwa, Roma Patel, Sayantani Pramanik Palit, Naisargi Patel, Satyashree Shetty, Mitesh Dwivedi, Rasheedunnisa Begum, A.V. Ramachandran

**Affiliations:** 1 Department of Biochemistry, Faculty of Science, The Maharaja Sayajirao University of Baroda, Vadodara, Gujarat, India; 2 C. G. Bhakta Institute of Biotechnology, Faculty of Science, Uka Tarsadia University, Surat, Gujarat, India; 3 Division of Life Science, School of Sciences, Navrachana University, Vadodara, Gujarat, India

**Keywords:** dyslipidemia, genotype-phenotype correlation, haplotype, leptin, single nucleotide polymorphism, type 2 diabetes

## Abstract

**Background & aim:**

Leptin (LEP), a pro-inflammatory adipokine secreted by adipocytes acting through leptin receptor (LEPR), is critical in maintaining body weight, lipid and glucose metabolism. *LEP* and *LEPR* genetic variants are reportedly associated with type 2 diabetes (T2D). Among these, the *LEPR rs1137101* A/G polymorphism has emerged as a potential determinant of metabolic risk. The current study investigates the association of *LEP* and *LEPR* genetic variants, along with their transcript and protein levels, and evaluates genotype-phenotype correlations with metabolic parameters and T2D susceptibility in the Gujarat population.

**Methods:**

Genomic DNA isolated from PBMCs of 451 controls and 439 patients was used for genotyping *LEP* (*rs7799039* G/A; *rs2167270* G/A) and *LEPR* (*rs1137101* A/G; *rs1805094* G/C) polymorphisms by PCR-RFLP. RNA isolated from PBMCs was used to assess *LEP* and *LEPR* transcript levels by qPCR. Fasting Blood Glucose (FBG) levels, Body Mass Index (BMI) and plasma lipid profile were also assessed for the genotype-phenotype correlation analysis. ELISA was performed to estimate plasma protein levels of leptin and its soluble receptor (sOb-R).

**Results:**

Our findings suggest a significant association of *LEPR rs1137101 A/G* with T2D, where the GG genotype and G allele conferred a 1.66- and 1.24-fold increased risk for the disease, respectively. The GG genotype also showed an association with increased FBG and TC levels. In addition, an increased GG haplotype frequency, increased *LEP* transcript and protein levels, and decreased *LEPR* transcript and protein levels were observed in T2D patients. Moreover, leptin protein levels showed a positive correlation with increased BMI and TG, while sOb-R protein levels showed a positive correlation with increased BMI, FBG, and TG levels.

**Conclusion:**

The *LEPR rs1137101 A/G* polymorphism, together with elevated leptin, and decreased sOb-R protein levels, may increase susceptibility to T2D in the Gujarat population.

## 1 Introduction

Leptin (LEP), a pro-inflammatory adipokine encoded by the *ob* gene located on chromosome 7q31.3, is associated with food intake and appetite, energy homeostasis, basal metabolism, and insulin secretion (1–4). In normal physiology, leptin enhances insulin sensitivity by suppressing hepatic gluconeogenesis, stimulating glucose uptake in skeletal muscle, and modulating pancreatic β-cell insulin secretion (5). Leptin levels are elevated in obesity and T2D and are positively correlated with total body fat, fasting insulin, FBG, HOMA-IR, and HbA1c (1, 3, 5–8). High leptin levels in obesity indicate a state of leptin resistance, implicating impaired leptin receptor sensitivity and action (9, 10). This resistance disrupts the leptin-insulin axis, resulting in increased hepatic glucose output, reduced peripheral glucose utilization, and exacerbated insulin resistance (11). At the molecular level, impaired leptin signaling is linked to inflammatory and inhibitory pathways that interfere with insulin action and β-cell function, thereby leptin resistance links excess adiposity with insulin resistance, β-cell dysfunction, dyslipidaemia, and systemic inflammation, positioning leptin as a critical mediator in the pathogenesis of T2D (9, 12–15). Besides its central and peripheral effects on different tissues, leptin can also exert multiple actions on peripheral blood mononuclear cells (PBMCs) (16). *In vitro* studies have shown that leptin can directly induce the expression of its receptors in PBMCs and thereby trigger an inflammatory response (17). Leptin mediates its biological effects through interaction with the leptin receptor (LEPR), a single-pass transmembrane protein of the class I cytokine receptor family. Located on chromosome 1p31, LEPR gene is broadly distributed in diverse tissues such as the adipose tissue, skeletal muscle, liver, pancreatic islets, immune cells and the brain. Genetic and functional studies of LEPR have highlighted its importance in energy balance, obesity, insulin resistance, metabolic syndrome (MetS), and T2D (18, 19). Leptin circulates in a free form or is bound to its soluble receptor (sOb-R) (20). The sOb-R is the cleaved product of the extracellular domain of a membrane-bound leptin receptor, and elevated concentrations indicate regulation of leptin signaling (5). In obese adults, high serum leptin levels can reduce sOb-R levels and are associated with leptin resistance, although the molecular mechanism is not yet fully understood (21).

Several *LEP* and *LEPR* gene polymorphisms have been studied in different populations for their potential association with serum leptin levels, obesity, T2D, and MetS (22, 23). Among these variants, the *LEP* (G-2548A *rs7799039* G/A; 5’UTR *rs2167270* G/A) and *LEPR* (exon 6 Q223R *rs1137101* A/G; exon 14 K656N *rs1805094* G/C) single nucleotide polymorphisms (SNPs) have been studied in detail in different populations (23). However, only a few studies have investigated these polymorphisms in the Indian population (22, 24–26) reporting associations with obesity, BMI and T2D.

T2D cases are rising and will be ~74.9 million by 2030 in India, Gujarat being the second-highest state (27, 28). We have previously reported an association of adiponectin, vaspin, omentin, resistin, IL1-β, and TNF-α genes and their polymorphisms with T2D in the Gujarat population (29–34). We hypothesize that functional polymorphisms in *LEP* and *LEPR* may contribute to altered leptin expression and signaling, thereby influencing metabolic traits and susceptibility to T2D. Thus, the present study explored *LEP* and *LEPR* polymorphisms, their transcript levels in PBMCs, plasma protein levels, and genotype-phenotype correlations with metabolic parameters and T2D susceptibility in the Gujarat population.

## 2 Materials and methods

### 2.1 Study population

The study was approved by the Institutional Ethical Committee for Human Research (IECHR: FS/IECHR/2016-9) and conducted as per the Helsinki Declaration. All participants were informed about the significance of the study, and written consent was obtained from both patients and age- and sex-matched control subjects. Patients diagnosed with type 2 diabetes (T2D) were recruited from diabetes awareness camps and had fasting blood glucose (FBG) levels >125 mg/dL, with no other known comorbidities. Healthy controls exhibited FBG <110 mg/dL and had no prior history of diabetes. The clinical characteristics of both groups, as shown in **Supplementary Table S2**, differed significantly between controls and T2D patients. Both obese and lean individuals were included in the study. Body mass index (BMI) was calculated as weight (kg)/height (m²), and participants were classified based on Indian-specific BMI cut-offs recommended by the WHO: BMI <23 kg/m² was considered lean and BMI ≥23 kg/m² was considered obese (35). The controls and T2D cohorts were further subdivided into lean and obese subgroups to evaluate the potential influence of adiposity on leptin and sOb-R levels.

Individuals with autoimmune diseases, cancer, or other major illnesses were excluded from the study. Additionally, visceral (omental) adipose tissue samples were obtained from individuals undergoing bariatric surgery. A total of 439 T2D patients (males/females) and 451 (males/females) controls between 30 and 67 years of age from the Gujarati population were enrolled for this study. The total sample size of 890 was determined using G*Power software (36) considering the effect size of 0.2 that achieved 91.9% statistical power for T2D patients and 92.7% statistical power for controls to detect the association of *LEP* and *LEPR* gene polymorphisms in the present study.

### 2.2 Anthropometric measurements, lipid profile, and nucleic acid (DNA & RNA) extraction

Body Mass Index (BMI) was determined from participants’ height and weight. Following a 12-hour overnight fast, 3 mL of venous blood was drawn into K_3_EDTA coated tubes (J. K. Diagnostics, Rajkot, India) to assess Fasting Blood Glucose (FBG), Total Cholesterol (TC), Triglycerides (TG), Low-Density Lipoprotein (LDL) and High-Density Lipoprotein (HDL) using commercially available kits (Reckon Diagnostics P. Ltd, Vadodara, India). LDL was calculated according to Friedewald formula (1972). Genomic DNA and total RNA were extracted from PBMCs as previously described (34).

### 2.3 Genotyping of *LEP* and *LEPR* polymorphisms by PCR-RFLP

Polymerase Chain Reaction-Restriction Fragment Length Polymorphism (PCR-RFLP) was used for genotyping the *LEP* (*rs7799039* G/A; *rs2167270* G/A) and *LEPR* (*rs1137101* A/G; *rs1805094* G/C) polymorphisms. The primers used for PCR are shown in **Supplementary Table S1**. The PCR reaction mixture composition and protocol were as described previously (34).

### 2.4 Estimation of *LEP* & *LEPR* transcript and protein levels

The primers used for assessing *LEP*, *LEPR*, and *GAPDH* transcript levels from PBMCs are shown in **Supplementary Table S1**; the protocols used were as described earlier (34). PBMCs were used for transcript analysis as they express functional *LEP* and *LEPR*, reflecting systemic immuno-metabolic interactions underlying chronic inflammation and insulin resistance in T2D. Plasma protein levels of leptin and sOb-R in patients and age and sex-matched controls were estimated by human leptin ELISA Kit (Ray Biotech., GA, USA) and sOb-R ELISA Kit (Elabscience, WH, China), respectively, as per the manufacturer’s protocols with a sensitivity of 2 pg/mL and 0.19 ng/mL. All the plasma estimations were duplicated to ensure a % coefficient of variation below 10%.

### 2.5 Statistical analyses

All statistical analyses were performed using GraphPad Prism version 6 (GraphPad Software Inc; San Diego, CA, USA).

#### 2.5.1 Genetic analyses

Hardy–Weinberg equilibrium (HWE) for all polymorphisms was tested in cases and controls using chi-square analysis of observed and expected genotype frequencies. Genotype and allele frequency distributions of *LEP* and *LEPR* polymorphisms were compared between cases and controls using chi-square tests with 2×2 contingency tables. Statistical significance was set at *p* < 0.025 after Bonferroni correction. The odds ratio (OR) for disease susceptibility was calculated with a 95% confidence interval (CI). Haplotypes and linkage disequilibrium (LD) coefficients D’ = D/Dmax and r^2^ values for the pair of the most common alleles at each site were obtained using online tool SHEsisPlus (37), a validated haplotype analysis platform based on the original SHEsis algorithm developed by Shi and He, which has been extensively applied in genetic association studies (38).

#### 2.5.2 Biochemical and expression analyses

Relative gene expression and fold change (2^-ΔΔCt^) of *LEP* and *LEPR*, and plasma protein levels of leptin and sOb-R in patient and control groups, were plotted and analyzed using an unpaired t-test. Associations between *LEP* and *LEPR* polymorphisms and anthropometric parameters were evaluated using analysis of variance (ANOVA) and the Kruskal–Wallis test.

#### 2.5.3 Correlation analyses

All correlation analyses were conducted employing multiple linear regression and Spearman’s correlation methods.

The level of statistical significance was determined using standardized thresholds of *p* < 0.05, *p* < 0.01, and *p* < 0.001, while values > 0.05 were considered non-significant (*p* > 0.05). For multiple comparisons where Bonferroni correction was applied, *p* < 0.025 was considered statistically significant.

### 2.6 Bioinformatics analyses

*In silico* prediction tools PANTHER (39), MUPRO (40), and I-MUTANT SUITE (41) were employed to predict the sequence-based impact of single amino acid variations on protein structure and stability.

## 3 Results

### 3.1 Clinical characteristics

The clinical characteristics differed significantly between controls and T2D patients, as shown in **Supplementary Table S2**.

### 3.2 Genetic associations

#### 3.2.1 Association of *LEP* and *LEPR* polymorphisms with T2D

The genotype and allele frequencies of the studied *LEP* and *LEPR* polymorphisms are summarized in **Table 1**. At the same time, the representative gel images for PCR-RFLP analysis are shown in **Supplementary Figure S1**. Confirmation of genotyping results by Sanger sequencing of PCR products for the respective polymorphisms is shown in **Supplementary Figure S2**. The distribution of genotype frequencies for all the investigated polymorphisms was consistent with Hardy-Weinberg expectations in both patient and control groups (*p* > 0.05).

**Table 1:** Distribution of genotype and allele frequencies of *LEP* and *LEPR* polymorphisms in T2D patients and controls.

SNP	Genotype/allele	Controls (Freq.)	Patients (Freq.)	*p*-value for HWE	*p* value	Odds ratio	CI (95%)
*LEP* -2548 rs7799039 (G/A)		n=297	n=297				
	GG	77 (0.26)	78 (0.26)	(C)	R	–	–
	GA	139 (0.47)	140 (0.47)	> 0.05	> 0.05^a^	0.99	0.67-1.47
	AA	81 (0.27)	79 (0.26)		> 0.05^b^	0.96	0.61-1.49
				(P)			
	G	293 (0.49)	296 (0.50)	> 0.05	R	–	–
	A	301 (0.51)	298 (0.50)		> 0.05^c^	0.98	0.78-1.23
*LEP* 5’UTR rs2167270 (G/A)		n=269	n=268				
	GG	140 (0.52)	138 (0.51)	(C)	R	–	–
	GA	106 (0.39)	116 (0.43)	> 0.05	> 0.05^a^	1.11	0.79-1.58
	AA	23 (0.08)	14 (0.05)		> 0.05^b^	0.61	0.30-1.25
				(P)			
	G	386 (0.72)	392 (0.73)	> 0.05	R	–	–
	A	152 (0.28)	144 (0.27)		> 0.05^c^	0.93	0.71-1.21
*LEPR* Exon 6 Q223R rs1137101 (A/G)		n=451	n=439				
	AA	143 (0.31)	125 (0.28)	(C)	R	–	–
	AG	238 (0.53)	212 (0.48)	> 0.05	> 0.05^a^	1.01	0.75-1.38
	GG	70 (0.16)	102 (0.23)		**< 0.01^b^**	1.66	1.13-2.45
				(P)			
	A	524 (0.58)	462 (0.53)	> 0.05	R	–	–
	G	378 (0.42)	416 (0.47)		**< 0.025^c^**	1.24	1.03-1.5
*LEPR* Exon 14 K656N rs1805094 (G/C)		n=302	n=308				
	GG	260 (0.86)	250 (0.81)	(C)	R	–	–
	GC	42 (0.14)	58 (0.19)	> 0.05	> 0.05^a^	1.43	0.93-2.21
	CC	0	0		–	–	–
				(P)			
	G	562 (0.93)	558 (0.91)	> 0.05	R	–	–
	C	42 (0.07)	58 (0.09)		> 0.05^c^	1.39	0.91-2.10

‘n’, number of samples; ‘R’, reference group; CI, confidence interval. a, b: genotype comparisons (patients vs controls); c: allele comparisons (patients vs controls) both using the chi-square test with 2×2 contingency table. Statistical significance was set at *p* < 0.025 after Bonferroni correction.

Bold values indicate statistically significant differences.

The genotype and allelic frequencies of *LEP rs7799039* G/A and *rs2167270* G/A polymorphisms and *LEPR rs1805094* G/C polymorphism were found to be statistically non-significant (*p* > 0.05), and therefore were not analyzed further after the initial assessment. However, the GG genotype (*p <* 0.01) and mutant allele ‘G’ (*p <* 0.025) of *LEPR rs1137101* A/G were associated with increased risk for T2D with an Odds Ratio (OR) of 1.66 and 1.24, respectively, with modest effect sizes (**Table 1**).

#### 3.2.2 Haplotype and linkage disequilibrium analysis

A haplotype analysis of *LEPR rs1137101* A/G and *rs1805094* G/C polymorphic site revealed significant differences between patients and controls (global *p <* 0.05), and the susceptible disease haplotype is GG (*p <* 0.05) (**Table 2**). Furthermore, the LD analysis revealed that two polymorphic sites of the *LEPR* gene had a low LD association (D’=0.723, r2 = 0.038), as shown in **Supplementary Figure S3**. Although statistically significant, the odds ratios associated with the *LEPR rs1137101* GG genotype and the GG haplotype indicate a modest effect size, consistent with the polygenic nature of T2D.

**Table 2:** Distribution of haplotype frequencies of *LEPR* polymorphisms in T2D patients and controls.

Haplotype (*LEPR* rs1137101, rs1805094)	Patients (Freq. %) (n=275)	Controls (Freq. %) (n=300)	*p* for association	*P* (Global)	Odds ratio	95% CI
A C	43.74 (0.080)	33.91 (0.070)	0.497	**< 0.05**	1.17	0.73-1.8
**A G**	240.26 (0.440)	257.09 (0.531)	< 0.01		**0.71**	0.55-0.91
G C	8.26 (0.015)	0.09 (0.000)	–		–	–
**G G**	253.74 (0.465)	192.91 (0.399)	**< 0.05**		**1.35**	1.05-1.72

CI’, confidence interval (frequency < 0.03 in patients and controls were excluded from the analysis). Bold values indicate statistically significant differences.

### 3.3 Functional consequences of LEP and LEPR dysregulation

#### 3.3.1 Assessment of *LEP* and *LEPR* transcript levels from PBMCs

**Figure 1:**
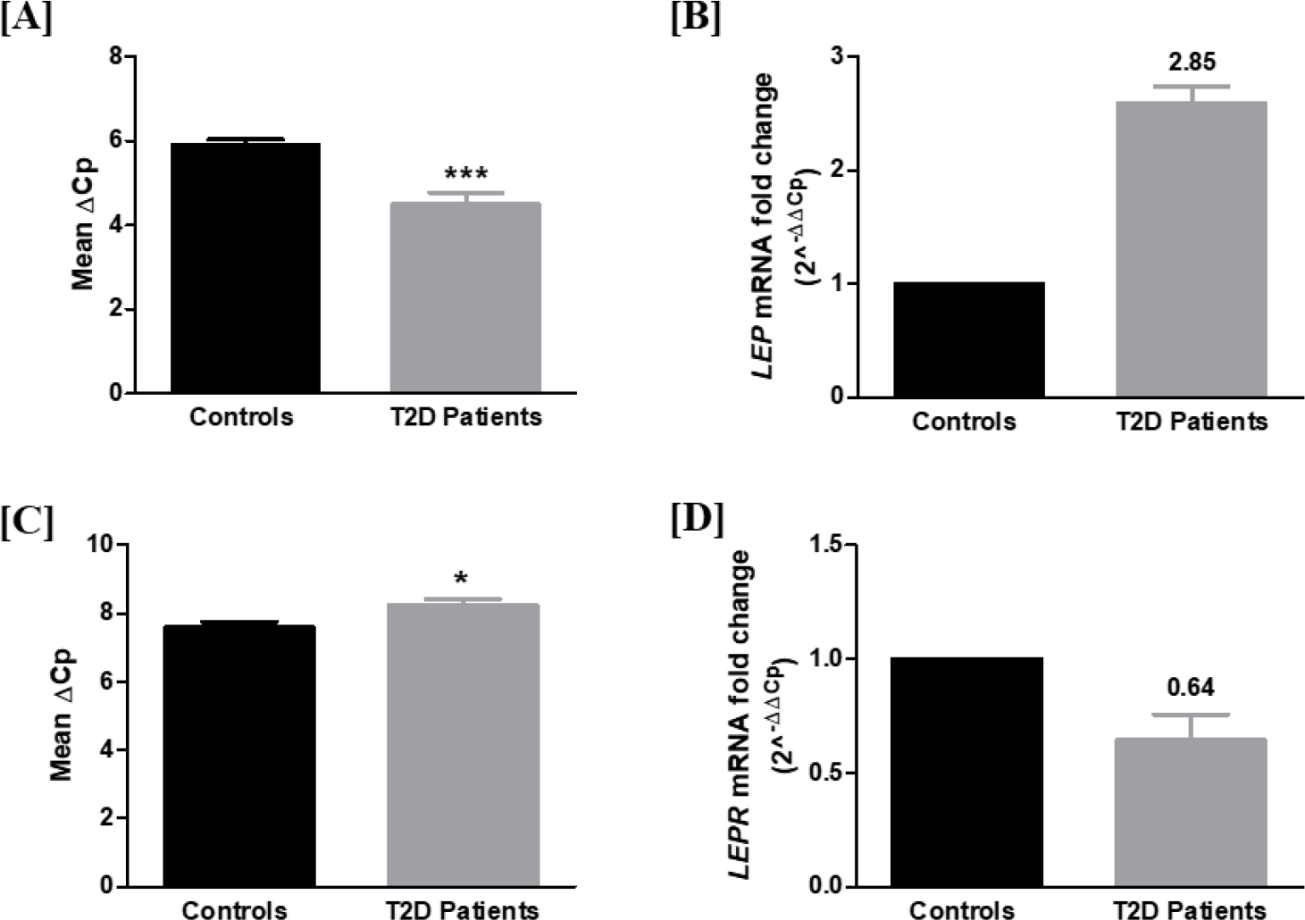
*LEP* and *LEPR* transcript levels in PBMCs of T2D patients and controls. **(A)** Relative gene expression of *LEP* in PBMCs of controls and patients: A significant increase in *LEP* transcript was observed in patients (Mean ΔCp ± SEM: 5.88 ± 0.143 vs 4.5 ± 0.26; *p* < 0.001). **(B)** Relative fold change of *LEP* expression in controls and patients: Diabetic patients showed a 2.85-fold increase in *LEP* mRNA expression as determined by the 2^-ΔΔCp^ method (controls n=120; T2D patients n=119). **(C)** Relative gene expression of *LEPR* in PBMCs of controls and patients: A significant decrease in *LEPR* mRNA transcript was observed in patients (Mean ΔCp ± SEM: 7.58 ± 0.17 vs 8.22 ± 0.19; *p* < 0.05). **(D)** Relative fold change of *LEPR* expression in controls and patients: Diabetic patients showed a 36% reduction in *LEPR* mRNA expression as determined by the 2^-ΔΔCp^ method (controls n=179; T2D patients n=168). * indicates p < 0.05, and *** indicates p < 0.001.

*LEP* transcript levels were significantly increased in PBMCs of 119 patients compared with 120 controls, after normalization with *GAPDH* expression, as indicated by mean ΔCp values (*p* < 0.001) with a fold change of 2.85 (**Figures 1A, B**).

Further, *LEPR* transcript levels were found to be significantly decreased (36% reduction) in PBMCs of 168 patients compared with 179 controls, as suggested by mean ΔCp values (*p* < 0.05) with a fold change of 0.64 (**Figures 1C, D**).

#### 3.3.2 Estimation of plasma protein levels of leptin and sOb-R

**Figure 2:**
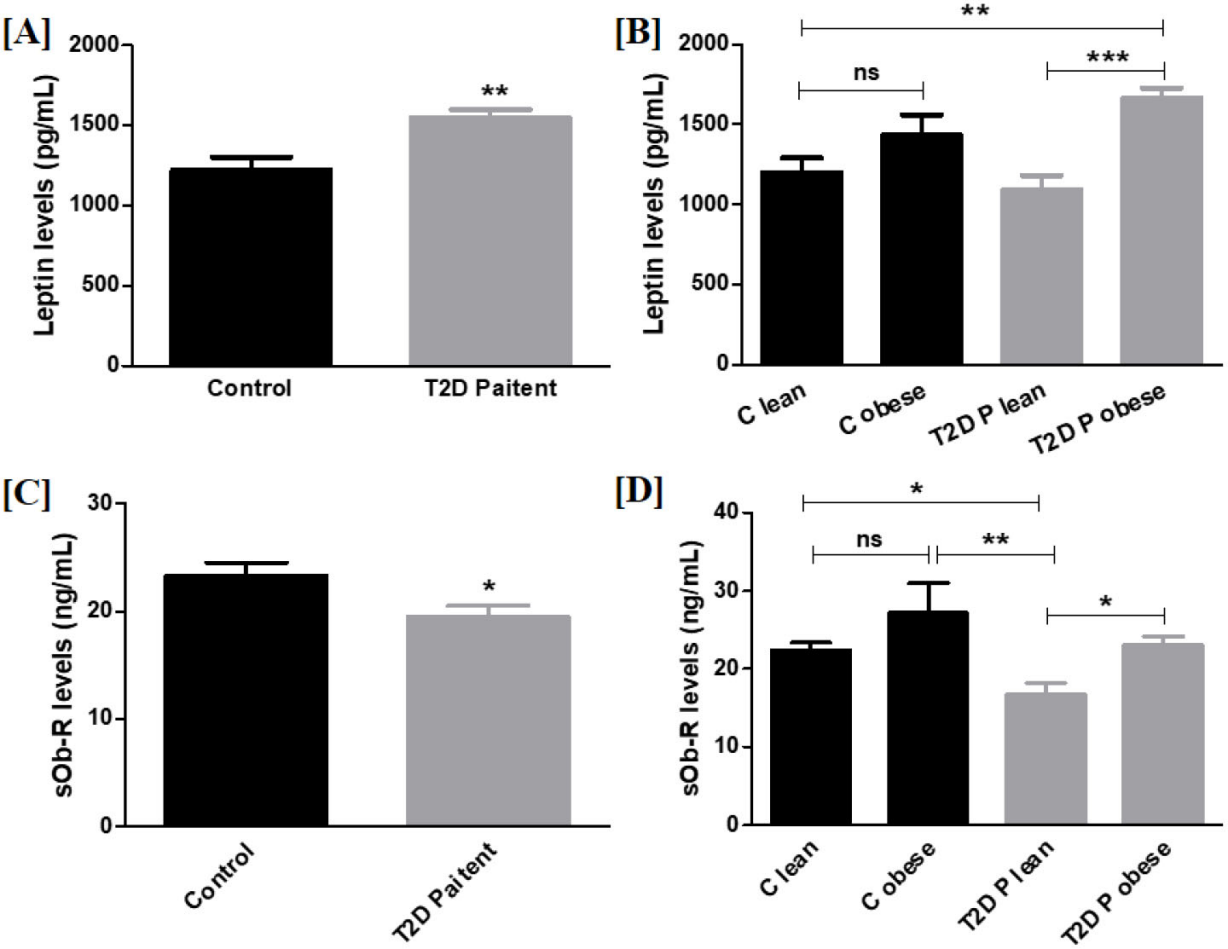
Plasma protein levels of leptin and sOb-R in T2D patients and controls. **(A)** Plasma leptin protein levels in controls vs patients: leptin levels were considerably increased in patients (*p* < 0.01). **(B)** Control lean vs obese and patient lean vs obese: control lean vs patient obese (*p* < 0.01) and patient lean vs obese (*p* < 0.001) showed a considerable difference, while no difference was observed between other groups. **(C)** Plasma sOb-R levels in controls vs. patients: sOb-R levels were considerably decreased in patients (*p* < 0.05). **(D)** Control lean vs obese and patient lean vs obese: Control lean vs obese lean (*p* < 0.05), control obese vs patient lean (*p* < 0.01), and patient lean vs obese (*p* < 0.05) showed significant differences (controls n=44; T2D patients n=42). * indicates p < 0.05; ** indicates p < 0.01, and *** indicates p < 0.001.

Plasma leptin and sOb-R protein levels were assessed in 44 controls and 42 patients. Plasma leptin levels were significantly elevated in T2D patients compared with controls (*p* < 0.01), and particularly in obese patients relative to lean patients (*p* < 0.001) and lean controls (*p* < 0.01) (**Figures 2A, B**). In addition, sOb-R levels were markedly reduced in T2D patients compared with controls (*p* < 0.05), as shown in **Figure 2C**. Interestingly, we found a significant elevation in sOb-R levels in obese patients as compared with lean patients (*p* < 0.05) and reduced sOb-R levels in lean patients compared with lean (*p* < 0.05) and obese controls (*p* < 0.01) (**Figure 2D**).

### 3.4 *LEP* and *LEPR* correlations with metabolic characteristics

#### 3.4.1 Correlation of *LEP* and *LEPR* polymorphisms with FBG, BMI and plasma lipids

Correlation analysis revealed no significant association between *LEP* (*rs7799039 G/A, rs2167270 G/A*) and *LEPR* (*rs1805094 G/C*) polymorphisms and FBG, BMI, or plasma lipids (*p* > 0.05). In contrast, the GG genotype of *LEPR rs1137101* A/G polymorphism was significantly associated with elevated FBG (*p <* 0.05) and TC (*p <* 0.05) levels, but showed no significant association with BMI and plasma lipid profile, i.e., TG, LDL and HDL (**Table 3**).

**Table 3:** Genotype-phenotype correlation of *LEP* and *LEPR* polymorphisms with BMI, FBG and plasma lipid profile.

Genotype	FBG (mg/dl)	BMI (kg/m^2^)	TG (mg/dl)	TC (mg/dl)	LDL (mg/dl)	HDL (mg/dl)
*LEP* -2548 G/A (rs7799039)						
GG	160.5 ± 27.73	28.48 ± 2.59	221.8 ± 64.2	170.1 ± 35.75	98.44 ± 29	37.36 ± 11.47
GA	190 ± 66.75	29.98 ± 3.74	216 ± 56.95	174.6 ± 39.67	106.3 ± 32.52	36.75 ± 12.56
AA	176 ± 75.59	29.52 ± 3.49	210 ± 58.20	170.3 ± 30.26	103.8 ± 29.77	37.81 ± 14.26
*P* value	> 0.05	> 0.05	> 0.05	> 0.05	> 0.05	> 0.05
*LEP* 5’UTR G/A (rs2167270)						
GG	151.6 ± 62.32	27.74 ± 5.634	161.6 ± 80.14	173.7 ± 43.25	103.4 ± 39.29	40.49 ± 12.3
GA	144.6 ± 50.76	28.27 ± 5.814	151.8 ± 74.05	171.6 ± 47.71	106.9 ± 34.60	40.54 ± 15.01
AA	128.6 ± 20.35	28.13 ± 5.600	181.9 ± 79.36	170.9 ± 26.02	87.28 ± 31.14	47.19 ± 20.34
*P* value	> 0.05	> 0.05	> 0.05	> 0.05	> 0.05	> 0.05
*LEPR* Q223R Exon 6 A/G (rs1137101)						
AA	150.3 ± 38.13	29.85 ± 5.13	207.5 ± 44.3	182.5 ± 19.77	100.6 ± 32.7	37.34 ± 12
AG	158 ± 56.17	29.63 ± 3.6	210.4 ± 66.82	191.7 ± 26.24	107 ± 31.49	38.4 ± 14
GG	171.7 ± 48.51	29.39 ± 3.7	199.6 ± 35.64	194.6 ± 23.96	106.9 ± 28.03	38.56 ± 10.58
*P* value	**< 0.05**	> 0.05	> 0.05	**< 0.05**	> 0.05	> 0.05
*LEPR* K656N Exon 14 G/C (rs1805094)						
GG	151.1 ± 54.78	27.86 ± 6.531	162.7 ± 82.51	166.5 ± 35.71	104.6 ± 37.21	41 ± 14.05
GC	148.1 ± 66.23	27.83 ± 5.595	160.2 ± 71.10	175.0 ± 40.75	111.6 ± 35.81	45.12 ± 17.16
*P* value	> 0.05	> 0.05	> 0.05	> 0.05	> 0.05	> 0.05

Data are presented as Mean ± SD. Statistical significance was considered at *p* < 0.05.

Bold values indicate statistically significant differences.

#### 3.4.2 Plasma protein levels of leptin and sOb-R and their correlation with metabolic profile

Spearman’s correlation analysis revealed that leptin was positively correlated with BMI (*p* < 0.001) and TG (*p* < 0.05), while sOb-R protein levels were positively correlated with BMI (*p* < 0.001), FBG (*p* < 0.05), and TG (*p* < 0.01) (**Table 4**).

**Table 4:** Correlation analysis of leptin and sOb-R plasma protein levels with the metabolic profile.

Parameters	Leptin		sOb-R	
	*r^2^*	*p*	*r^2^*	*P*
BMI (Kg/m^2^)	0.5224	**< 0.001**	0.3941	**< 0.001**
FBG (mg/dL)	0.0222	> 0.05	0.1214	**< 0.05**
Triglycerides (mg/dL)	0.1391	**< 0.05**	0.1574	**< 0.01**
Total Cholesterol (mg/dL)	0.0002	> 0.05	0.0126	> 0.05
HDL (mg/dL)	0.0014	> 0.05	0.0001	> 0.05
LDL (mg/dL)	0.0047	> 0.05	0.0055	> 0.05

R, Spearman’s correlation Coefficient [*p* < 0.05, significant; *p* > 0.05, non-significant]. Bold values indicate statistically significant differences.

### 3.5 Bioinformatics analysis

Using bioinformatics tools, we further investigated the impact of *LEPR* rs1137101 polymorphism on its protein function. This polymorphism results in a Glutamine to Arginine substitution at position 223 (Gln223Arg). PANTHER tool showed that Gln223Arg variation is probably damaging to *LEPR* function. I-MUTANT and MUPRO predictions revealed decreased stability for the Gln223Arg *LEPR* variant compared with the native structure (**Table 5**).

**Table 5:** *In-silico* prediction results for *LEPR* Q223R A/G polymorphism.

Amino acid change	PANTHER	I-MUTANT	MUPRO
Gln223Arg	Probably Damaging	Decreased stability	Decreased stability

## 4 Discussion

The *LEP* and *LEPR* genes has been widely reported across various populations as key loci associated with T2D, with several single nucleotide polymorphisms identified as potential genetic risk factors (23). Our study is the first to evaluate the association between *LEP* and *LEPR* polymorphisms and T2D in the Gujarat population. Our results revealed no association between *LEP* (*rs7799039* G/A and *rs2167270* G/A) and *LEPR* (*rs1805094* G/C) polymorphisms and T2D risk or any anthropometric parameters. Similar observations were reported in Egyptian, Turkish, Polish, Iranian, Swiss, Mexican, and North and South Indian populations (22, 26, 42–47). We report a significant association for *LEPR rs1137101* A/G polymorphism with T2D; the GG genotype and G allele show a 1.66- and 1.24-fold increased risk for T2D, respectively. Moreover, the GG genotype was strongly correlated with elevated FBG and TC levels. Similarly, Boumaiza et al. (2012) showed an association of this polymorphism with TC and BMI in the Tunisian population (48). However, similar to our findings, studies in Turkish (49) and Brazilian (50) populations showed no association between *LEPR rs1137101* A/G polymorphism and BMI.

Several case-control studies across Malaysian, Indian (Punjabi and Coimbatore), Korean, Chinese, Malay, Slavonic, and Ukrainian populations have reported positive associations between *LEPR rs1137101* and T2D risk (22, 26, 51–55). This polymorphism causes a non-conservative substitution within the extracellular leptin-binding domain (N-terminal CRH1 domain), by converting glutamine to arginine at codon 223 (CAG to CGG), which leads to a change in the charge from neutral to positive. Thus, this amino acid change affects all isoforms of the receptor and may be linked to impaired signal transduction, thereby increasing susceptibility to T2D (56–58). Our *in silico* analyses are consistent with this interpretation, suggesting that variation probably damages LEPR function and decreases the stability of the mutant LEPR protein.

*LEP* and *LEPR* expression in PBMCs and plasma sOb-R protein levels of T2D patients and controls were assessed for the first time in the Gujarat population. T2D patients showed a 2.85-fold increase in *LEP* transcript and plasma leptin protein levels. Further, T2D patients showed a 36% reduction in *LEPR* transcript and reduced plasma sOb-R protein levels. According to several studies, hyperleptinemia and decreased sOb-R protein levels are key markers of leptin resistance (59, 60). We found a strong, inverse association between circulating sOb-R protein levels and the risk of T2D. Sun et al. (2010) reported a similar observation in the U.S. population (61). Our correlation analysis reveal that plasma leptin levels significantly correlate with BMI and TG levels, which aligns with these findings. In contrast, plasma sOb-R protein levels significantly correlate with BMI, FBG and TG levels.

In our population, plasma leptin and sOb-R levels increase in obese T2D patients compared to lean T2D patients. These results are in line with previous findings (62). However, a contradictory study has reported reduced sOb-R protein levels associated with obesity. This study suggested that increased sOb-R protein levels might augment leptin’s physiological actions in lean subjects compared to obese subjects (21). Devos et al. (1997) suggested that complexes of leptin with sOb-R reflect a molecular ratio 1:1 (63). Two-fold or higher levels of circulating sOb-R suppressed leptin action *in vitro* and *in vivo* (59). This study indicated that increased sOb-R protein levels could be one of the reasons for leptin resistance in obesity.

The alarmingly rising prevalence of obesity poses adverse health problems (64, 65). Genetic and environmental factors make it a multifactorial and heterogeneous condition (66, 67). It is well known that obesity can lead to T2D, especially when characterized by insulin resistance (68). Chronic low-grade inflammation is a trademark of obesity, resulting from a disruption in the balance between pro- and anti-inflammatory adipokines (69). A pro-inflammatory adipokine resistin is reported to be elevated in T2D (70) and is known to activate cAMP-mediated PKA and NF-kB signaling pathways, promoting the expression of various inflammatory adipokines, including TNF-α and IL-1β (71). Moreover, hyperleptinemia can trigger an inflammatory response in PBMCs and promote TNF-α and iNOS synthesis by activating the JAK/STAT-3 pathway (72). We have reported a similar pattern of imbalance in pro- and anti-inflammatory adipokines, i.e., increased levels of pro-inflammatory resistin (32), TNF-α (34), and IL-1β (33) and reduced levels of anti-inflammatory adiponectin (29), vaspin (30) and omentin-1 (31) in T2D patients of the Gujarat population.

Previously, we also reported a significant association of angiotensin-converting enzyme (ACE) I/D polymorphism with T2D in the Gujarat population (73). The ACE ‘D’ allele was associated with elevated angiotensin II levels (74), and studies have suggested that increased angiotensin II leads to decreased adiponectin levels. ACE plays a crucial role in glucose metabolism through the renin–angiotensin system (RAS). Elevated ACE activity increases angiotensin II production, which in turn promotes oxidative stress, endothelial dysfunction, and impaired insulin signaling, collectively contributing to insulin resistance and T2D development (75). This mechanistic relationship has also been supported by recent evidence, as highlighted in a meta-analysis by Yao et al. (2021), demonstrating that ACE inhibition may confer additional benefits in improving insulin resistance (76). Our findings, in line with previous studies, suggest that altered ACE may promote T2D susceptibility through RAS-driven metabolic and inflammatory dysregulation. Furthermore, circadian rhythms play a crucial role in regulating adipose tissue metabolism as well as the expression and secretion of adipokines (77, 78). This regulation is believed to be mediated by melatonin, a pineal gland hormone, acting on receptors in visceral adipose tissue or via the sympathetic nervous system (79, 80). Disruption in melatonin synthesis or receptor signaling can negatively affect insulin sensitivity and β-cell function, which may contribute to T2D risk (81–84). Genetic variants in the *MTNR1B* gene, particularly the rs10830963 G allele, are associated with elevated fasting glucose, impaired insulin secretion, and increased risk of T2D (85, 86). Our earlier study reported decreased plasma melatonin levels in individuals with T2D (87). We also observed that a neuropeptide Y (NPY) promoter polymorphism regulates NPY levels, which in turn reduces melatonin levels (33). Impaired melatonin signaling leads to leptin resistance, highlighting its critical role in leptin signaling and regulation (88). Melatonin helps maintain the expression and secretion of leptin and adiponectin (89), offering insight into its broader relevance to obesity. Hyperleptinemia and increased TNF-α levels could disrupt the balance between pro-inflammatory/anti-inflammatory adipokines along with melatonin levels, which could play a major role in the development of leptin and insulin resistance, thereby increasing the risk of obesity-induced T2D in the Gujarat population.

**Figure 3:**
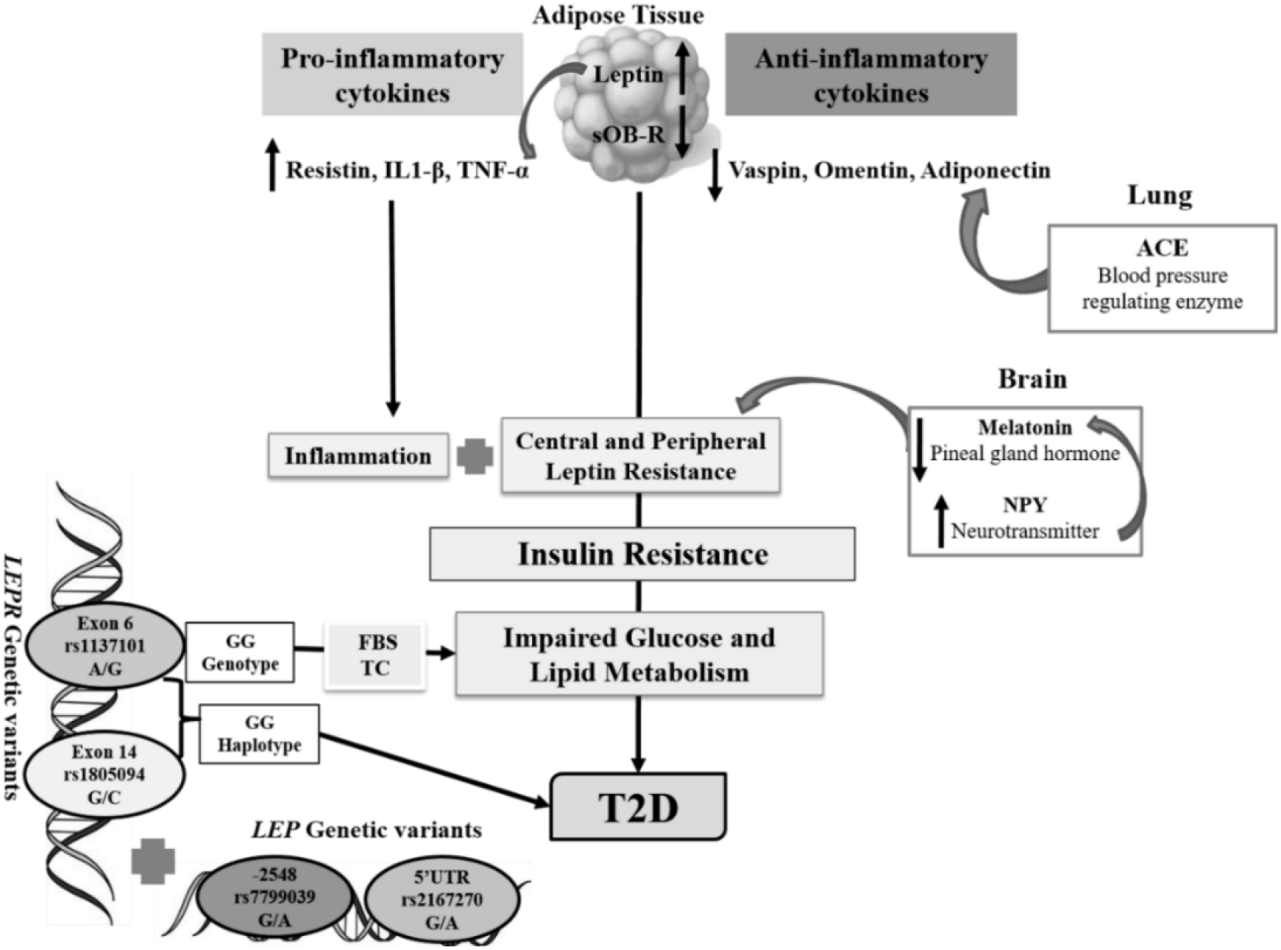
Role of leptin (LEP) and leptin receptor (LEPR, sOb-R) and altered adipokine levels in T2D. *LEPR rs1137101* A/G polymorphism is significantly associated with T2D, with the GG genotype increases disease risk by 1.66-fold. Moreover, the GG genotype strongly correlates with elevated FBG and TC levels. A haplotype evaluation of *LEPR rs1137101* A/G and *rs1805094* G/C further revealed that the GG haplotype is associated with T2D and increases the risk of diseases by 1.35 fold. The elevated leptin and reduced sOb-R levels may contribute to leptin resistance. Hyperleptinemia further elevates TNF-α levels, which play an important role in disrupting the balance between pro-inflammatory (resistin, IL1-β, TNF-α)/anti-inflammatory (vaspin, adiponectin, and omentin) adipokines and melatonin levels. Further, ACE and NPY down-regulate the anti-inflammatory adipokine- adiponectin and melatonin, respectively.

A summary illustrating the potential impact of *LEP & LEPR* polymorphisms and their altered transcript and protein levels, along with altered adipokines in obesity-induced T2D, is shown in **Figure 3**.

## 5 Implications, limitations, and future recommendations

The present findings deepen our understanding of the Leptin-sOb-R Axis in metabolic regulation and highlight a molecular link between obesity-induced inflammation and insulin resistance. By identifying associations between *LEPR* gene variants and altered leptin signaling components, this study strengthens the conceptual framework connecting genetic predisposition, leptin resistance, and chronic inflammation in T2D. Practically, these findings add valuable population-specific genetic data to the limited evidence available for Indian cohorts. Given the ethnic diversity within India, identifying genetic variants associated with metabolic risk in the Gujarati population provides a foundation for developing personalized medicine and public health strategies. These findings may support the future development personalized risk prediction, early screening strategies, and targeted lifestyle or pharmacological interventions.

Despite providing valuable insights, the present study has certain limitations. Although the sample size was adequate to detect significant associations, expanding the cohort to include additional regions of Gujarat and other Indian states would improve generalizability. While this study assessed *LEP*/*LEPR* polymorphisms and corresponding transcript and protein levels, functional validation (e.g., luciferase reporter or promoter assays) was not performed. This gap was partially addressed by *in silico* analyses. Environmental and lifestyle factors (e.g. diet, physical activity, and socioeconomic status) were not extensively examined. Furthermore, this study did not evaluate associations between *LEP*/*LEPR* polymorphisms and other circulating adipokines (adiponectin, vaspin, omentin, resistin, IL-1β, TNF-α) limiting insights into gene-adipokine interactions that may contribute to diabetes risk.

Future studies should include larger multi-ethnic cohorts across India to validate these findings and examine gene-environment interations. Experimental functional assays are necessary to confirm regulatory effects of *LEP* and *LEPR* polymorphisms on gene expression. Further, integrating *in vitro*/*in vivo* models with *in silico* predictions and multi-omics approaches will help define the mechanistic consequences of leptin-LEPR dysregulation. Finally, longitudinal or intervention-based studies assessing how lifestyle modification, diet, or pharmacological agents influence leptin sensitivity in genetically predisposed individuals may guide personalized diabetes prevention and management strategies.

## 6 Conclusions

This study is the first to identify an association between the *LEPR rs1137101* A/G polymorphism and T2D in the Gujarat population, with the GG genotype conferring a 1.66-fold higher susceptibitlity and correlating with elevated FBG and TC levels. Additionally, increased leptin and reduced sOb-R levels observed in T2D patients indicate a potential contribution to leptin resistance. Integrating these results with our previously published work on adipokines, a mechanistic framework emerges wherein hyperleptinemia may promote elevated TNF-α and related pro-inflammatory adipokines, thereby disturbing the balance between pro- (resistin, IL1-β, TNF-α) and anti-inflammatory (vaspin, adiponectin, and omentin) adipokines as well as melatonin signaling. Such dysregulation could play a major role in the development of leptin and insulin resistance, ultimately increasing the risk of obesity-induced T2D in the Gujarat population. Thus, our findings provide new insights into the role of *LEP & LEPR* in leptin resistance-mediated T2D susceptibility.

## Data Availability

The original contributions presented in the study are included in the article/**Supplementary Material**. Further inquiries can be directed to the corresponding author.
