# Supplementary material for: Leptin receptor rs1137101 polymorphism and altered leptin-sOb-R axis contribute to type 2 diabetes risk in Gujarat population
Source: Front Endocrinol (Lausanne). 2026 Jan 28;17:1693265. doi: 10.3389/fendo.2026.1693265 (PMC12890640; doi:10.3389/fendo.2026.1693265)
Supplement: Supplementary file 1 [file DataSheet1.docx]

**Supporting Information:**

**Figure S1: [A]** PCR-RFLP analysis of *LEP* -2548 G/A polymorphism on 3.5% agarose gel: PCR product was digested by restriction enzyme *Hha1*. Digestion resulted in a 313-bp fragment for the A allele and 241 and 72 bp fragments for the G allele. **[B]** PCR-RFLP analysis of LEP 5’UTR G/A polymorphism on 3.5% agarose gel: PCR product was digested by restriction enzyme *HpyCH4III*. Digestion resulted in a 245bp fragment for the G allele and 183 and 62 bp fragments for the A allele. **[C]** PCR-RFLP analysis of LEPR Q223R Intron 6 A/G polymorphism on 3.5% agarose gel: PCR product was digested by restriction enzyme *MSPI*. Digestion resulted in a 277-bp fragment for the A allele and 189 and 88 bp fragments for the G allele. **[D]** PCR-RFLP analysis of LEPR K656N Intron 6 G/C polymorphism on 3.5% agarose gel: PCR product was digested by restriction enzyme *BstUI*. Digestion resulted in a 159-bp fragment for the G allele and 136 and 23 bp fragments for the C allele.


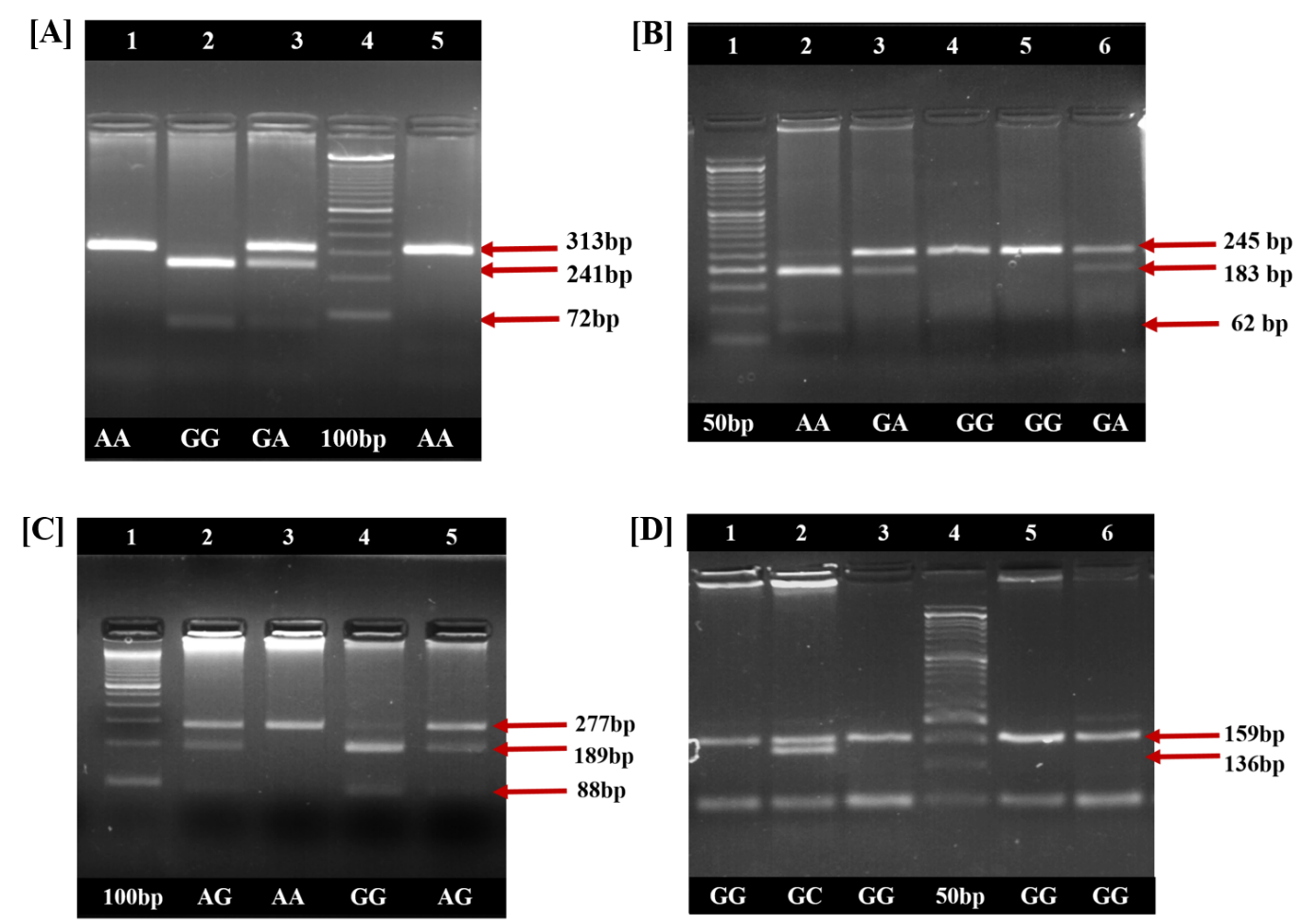


**Figure S2: Confirmation of genotyping results by Sanger’s sequencing of PCR products.** [A] *LEP* -2548 rs7799039 G/A Polymorphism. [B] *LEP* 5’ UTR rs2167270 G/A Polymorphism. [C] *LEPR* Q223R rs1137101 A/G Polymorphism. [D] *LEPR* K656N rs1805094 G/C Polymorphism.


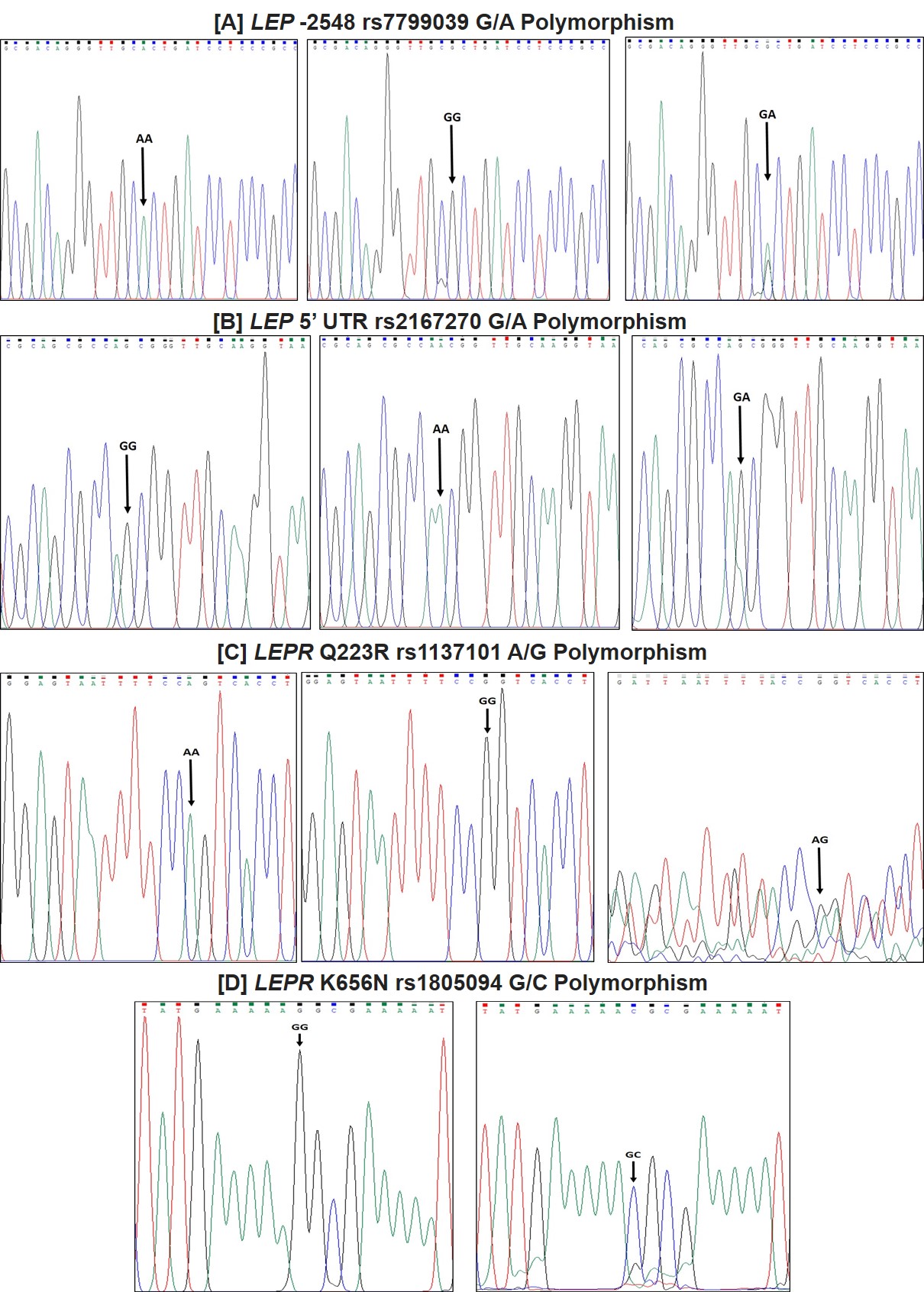


**Figure S3:** Linkage disequilibrium (LD) block: LD block with respect to *LEPR* *rs1137101* A/G and *rs1805094* G/C polymorphisms in Gujarat population.


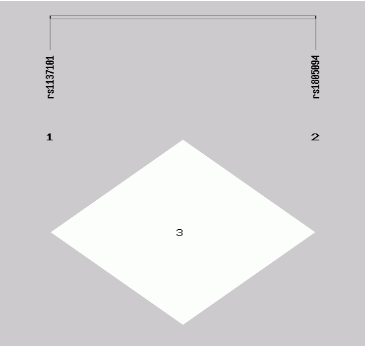


**Table S1:** Primers and restriction enzymes used for genotyping for *LEP* and *LEPR* polymorphisms and expression.

| **SNP/Gene Primer** | **Sequence (5'-3')** | **AT** | **Amplicon**  **Size (bp)** | **R.E.** | **Digested products size (bp)** |
| --- | --- | --- | --- | --- | --- |
| *LEP* -2548  G/A (rs7799039) | **FP:**5’GTGTGTTCCCTGGTTCAAGG3’  **RP:**5’GATCTCTCTGTTCGGGGTC3’ | 65°C | 313 | *HhaI* | 241 + 72 |
| *LEP* 5’UTR  G/A (rs2167270) | **FP:**5’GATCGGGCCGCTATAAGAG3’  **RP:**5’CCGGTAACCTTCTATCTGGC3’ | 62°C | 245 | *HpyCH4III* | 183 + 62 |
| *LEPR*  Q223R A/G (rs1137101) | **FP:**5’GTGAATGTCTTGTGCCTGTGC3’  **RP:**5’AGAAGCCACTCTTAATACCCCC3’ | 69°C | 277 | *MspI* | 189 + 88 |
| *LEPR*  K656N  G/C (rs1805094) | **FP:**5’GAAAGTGCATAAGTGTGTGCTTC3’  **RP:**5’CCAAAGTAAAGTGACATTTTTCGC 3’ | 61°C | 159 | *BstUI* | 136 + 23 |
| *Leptin* | **FP:**5’TCACACACGCAGTCAGTCTC3’  **RP:**5’GGATCACGTTTCTGGAAGGCA3’ | 68°C | 151bp | *-* | - |
| *Leptin receptor* | **FP:**5’TGTTCTGCCTGAAGTGTTAG3’  **RP:**5’AGAGTGTCGTTGAGTTTGG3’ | 61°C | 138bp | *-* | - |
| *GAPDH* | **FP:**5’CATCACCATCTTCCAGGAGCGAG3’  **RP:**5’CCTGCAAATGAGCCCCAGCCT3’ | 69°C | 122bp | *-* | - |

FP: Forward Primer; RP: Reverse Primer; bp: base pair; AT: annealing temperature; RE: restriction enzyme

**Table S2:** Baseline characteristics of diabetic and non-diabetic individuals from Gujarat population.

|  | **Controls**  **(Mean±SD)** |  | **Patients**  **(Mean±SD)** | **P value** |
| --- | --- | --- | --- | --- |
| Age  Sex: Male  Female  Fasting blood glucose (mg/dL)  BMI (Kg/m^2^)  Total Cholesterol (mg/dL)  Triglycerides (mg/dL)  HDL (mg/dL)  LDL (mg/dL)  Onset age (Years)  Duration of disease (Years)  Family history | (n =502) |  | (n =478) |  |
|  | 39.64 ± 16.35 yr  251 (50%)  251 (50%)  100.1 ± 7.32  24.24 ± 5.2  160.9 ± 42.2  111.7 ± 60.90  42.79 ± 15.94  95.32 ± 41.79  NA  NA  NA |  | 55.99 ± 10.42 yr  213 (44.5%)  265 (55.5%)  155.3 ± 62.09  27.04 ± 5.1  166.2 ± 39.68  164.5 ± 111.1  38.2 ± 12.6  95.10 ± 37.52  50.65 ± 10.10  8.06 ± 7.3  64 (14%) | -  -  -  **< 0.001**  **< 0.001**  **< 0.05**  **< 0.001**  **< 0.001**  > 0.05  -  -  - |

Data are presented as Mean±SD. Statistical significance was considered at *p* < 0.05.
